# Identification of a subpopulation of long-term tumor-initiating cells in colon cancer

**DOI:** 10.1042/BSR20200437

**Published:** 2020-08-25

**Authors:** Linglong Peng, Yongfu Xiong, Rong Wang, Ling Xiang, He Zhou, Haitao Gu

**Affiliations:** 1 Department of Gastrointestinal Surgery, The Second Affiliated Hospital of Chongqing Medical University, Chongqing 400014, China; 2 Department of Hepatobiliary Surgery, The Affiliated Hospital of North Sichuan Medical College, Sichuan 637000, China; 3 The Cancer Center of the Fifth Affiliated Hospital of Sun Yat-sen University, Zhuhai 519000, China; 4 Medical Department, Chongqing Maternal and Child Health Care Hospital, Chongqing 400014, China; 5 Department of Gastrointestinal Surgery, The First Affiliated Hospital of Chongqing Medical University, Chongqing 400014, China

**Keywords:** colon cancer, extensive tumorigenic ability, Long-term tumor-initiating cells, metastasis

## Abstract

Long-term tumor-initiating cells (LT-TICs) are viewed as a quantifiable target for colon cancer therapy owing to their extensive self-renewal and tumorigenic and metastatic capacities. However, it is unknown which subpopulation of colon cancer cells contains LT-TICs. Here, based on the methods for isolating and identifying cancer stem cells (CSCs) and the functional features of LT-TICs, we aimed to identify a subpopulation of LT-TICs. Among the six cell lines assessed, our results showed that CD133 and CD44 coexpression was only detected in HCT116 and HT29 cell lines. In HCT116 and HT29 cells, CD133^+^CD44^+^ cells not only shared the extensive tumorigenic potential of LT-TICs but also functionally reproduced the behaviors of LT-TICs that drive tumor metastasis (TM) formation, suggesting that CD133^+^CD44^+^ cells are a typical representation of LT-TICs in colon cancer. Mechanistically, the enhanced capacity of CD133^+^CD44^+^ cells to drive metastasis involves the up-regulated expression of Wnt-, epithelial–mesenchymal transition (EMT)-, and metastasis-related genes in these cells. Additionally, CD133^+^CD44^+^ cells presented significant chemoresistance compared with corresponding nontumorigenic CD133^−^CD44^−^ cells following exposure to oxaliplatin (OXLP) or 5-fluorouracil (5-FU). Accordingly, CD133^+^CD44^+^ cells contained lower reactive oxygen species (ROS) levels than CD1133^−^CD44^−^ cells, and the low ROS levels in CD133^+^CD44^+^ cells were related to the enhancement of antioxidant defense systems. More importantly, CD133^+^CD44^+^ cells developed less DNA damage after exposure to chemotherapeutics than CD133^−^CD44^−^ cells. In conclusion, we identified a subpopulation of LT-TICs in colon cancer.

## Introduction

Colon cancer is a major threat to human health, and its incidence is increasing [1]. Although 70–80% of colon cancer can be radically excised, most patients experience advanced recurrence or metastasis and die within 5 years, despite receiving adjuvant chemotherapy and surgery treatment [2]. Tumor-initiating cells (TICs) or cancer stem cells (CSCs) have been viewed as one of the most important factors for limited treatment efficacy [3,4]. Previous studies have shown that colon cancer is a stem cell disease, and only this small subset of cells is endowed with differentiation and self-renewal capacity. TICs are functionally responsible for tumor recurrence, metastasis and chemoresistance, and have distinct heterogeneity [5–7]. More recently, Dieter et al., using a molecular tracking strategy, identified three types of TICs with specific roles in colon cancer initiation and metastasis that form a tumor stem cell hierarchy: delayed contributing (DC)-TICs, tumor transient amplifying cells (T-TACs), and extensively self-renewing long-term (LT)-TICs [8]. They suggested that LT-TICs maintained tumor formation in serial xenotransplants and drove metastasis *in vivo* [8]. Thus, to cure colon cancer efficiently, it is necessary to isolate and identify which subpopulation of CSCs are LT-TICs.

Currently, there is no special way to isolate LT-TICs. LT-TIC phenotypes, including extensive tumorigenic and metastatic features, provide a basis for us to isolate LT-TICs. In addition, since CSCs are currently isolated according to the expression of related markers and identified by functional arrays [9–11], we therefore hypothesize that LT-TIC populations can be enriched by the use of LT-TIC functional characteristics that facilitate extensive self-renewal and metastasis and by selecting cells according to the expression of special cell surface markers. CD133 alone is widely used for isolating colon CSCs, and purified CD133^+^ cells are tumorigenic according to serial xenograft assays in immunodeficient NOD/SCID mice [4]. Moreover, xenotransplantation of CD133^+^ cells leads to a tumor that closely resembles the original malignancy in terms of both morphology and CSC marker expression [5]. However, subsequent studies demonstrated that although CD133 is a useful prognostic indicator for assessing the risk of colon cancer metastasis, recurrence, and progression, it seems unlikely to contribute directly to the metastasis of colon cancer [12–14]. These findings suggest that it is not enough to isolate the LT-TIC subset only by the marker CD133 because of the lack of capacity of CD133^+^ cells to drive metastasis. CD44, an additional marker of colon CSCs, is a protein involved in cancer cell migration and matrix adhesion in response to a cellular microenvironment [9,15–17]. During the process of colon cancer metastasis, cancer cell survival in suspension requires lipid raft-associated CD44, and nuclear CD44/acetylated-STAT3 generates cells with properties of CSCs and the epithelial–mesenchymal transition (EMT) phenotype by transcriptional reprogramming, leading to drug resistance, tumor metastasis (TM), and a resulting poor prognosis [18]. Although CD44^+^ cells isolated from colon tissues present robust tumorigenicity in a xenograft model and higher clonal formation capacities *in vitro* [9,19], whether these cells display long-term tumorigenic potential is still unknown, and using CD44 alone to isolate LT-TICs seems irrational.

In our study, considering the functional features of CD133^+^ and CD44^+^ cells, we hypothesized that the combination of CD133 and CD44 might be an ideal model for isolating and identifying LT-TICs. The present study attempts to investigate the hypothesis that LT-TIC populations can be enriched in CD133^+^CD44^+^ cells by the use of the two critical functional characteristics of LT-TICs, which are extensive self-renewal and readily metastasizing.

## Materials and methods

### Cell culture

Authenticated human established colon cancer cell lines SW480, LOVO, HT29, SW620, HCT116, and CACO2 were purchased from the Cell Bank of Type Culture Collection (Shanghai, China). HT29 and HCT116 were maintained in McCoy’s 5a medium (Gibco, U.S.A.) medium supplemented with 10% fetal bovine serum (FBS). SW480 and SW620 were cultured in Leibovitz’s L-15 medium (Gibco, U.S.A.) with 10% FBS. CACO2 was maintained in Eagle’s Minimum Essential Medium (Gibco, U.S.A.) supplemented with 20% FBS. LOVO was cultured in Ham’s F-12K Medium (Gibco, U.S.A.) supplemented with 10% FBS. Cells were cultured at 37°C with 5% CO_2_.

### Isolation and identification of CD133^+^CD44^+^ and CD133^−^CD44^−^ cells

The coexpression of CD133 and CD44 in the above six cell lines was analyzed by flow cytometry. For this purpose, six cultured cell lines were trypsinized, washed, and resuspended in PBS for the preparation of single-cell suspensions. These samples were then stained with phycoerythrin (PE)-labeled anti-CD133 antibody (Miltenyi Biotech, Germany) and fluorescein isothiocyanate (FITC)-labeled anti-CD44 antibody (eBiosciences, U.S.A.) and analyzed using an FACSCalibur flow cytometer (BD Bioscience, U.S.A.). Mouse IgG1κ antibody conjugated to PE (Miltenyi Biotech, Germany) and rat IgG2bκ antibody conjugated to FITC (eBioscience, U.S.A.) were used as isotype controls. After flow cytometry analysis, HCT116 and HT29 cells were used for isolation of putative CD133^+^CD44^+^ CSCs by magnetic bead sorting using a magnetic activated cell sorting (MACS) microbead kit (Miltenyi Biotech, Germany). Similarly, single-cell suspensions of HCT116 and HT29 cells were prepared. In other experiments, single-cell suspensions from xenograft tumors were prepared by mechanical dissociation and enzymatically dissociated by 1-h incubation with collagenase type IV (Sigma–Aldrich, Germany) at 37°C. Dissociated tumor cells were then incubated with CD133 microbeads (Miltenyi Biotech, U.S.A.) for 20 min at 2–8°C, and CD133^+^ cells and CD133^−^ cells were successfully separated using MACS magnet and MS columns (Miltenyi Biotech, U.S.A.). Then, the isolated CD133^+^ cells were incubated with CD44 microbeads (Miltenyi Biotech, U.S.A.), followed by separation of the microbeads until CD133^+^CD44^+^ cells were enriched. For CD133^−^CD44^−^ cell isolation, CD133^−^ cells were used for depletion with CD44 antibody. Purity identification for positive and negative separation was performed by flow cytometry after magnetic bead separation. Three independent experiments were performed.

### Animal studies

The animal studies were conducted in accordance with the Guide for the Care and Use of Laboratory Animals of the National Institutes of Health. All animal experimental procedures in the present study were approved by the Ethics Committee of Chongqing Medical University. Animal studies were performed in animal experience center of Chongqing Medical University. For *in vivo* tumor growth assay and orthotropic metastasis assay, mice were anesthetized by intraperitoneal injection of 0.5% pentobarbital sodium (45 mg/kg). To generate subcutaneous tumors, single-cell suspensions with >90% survival rate were subcutaneously injected into the lateral wall of 4-week-old female BALB/c nude mice. Tumor growth conditions were observed and recorded every 1 week. Volume = width^2^ × length/2. Subcutaneous tumors were analyzed by histology or flow cytometry. For the orthotopic implantation model, laparotomy was performed to exteriorize the cecum of mice. Isolated cells were suspended in 30 µl of DMEM F-12 medium and 20 µl Matrigel and injected into the cecal wall. Node mice were killed by decapition at the end of experiment.

### Western blot analysis

Cells were lysed in lysis buffer (1% NP-40, 50 mM Tris, 0.1% SDS, 1 mM PMSF, 10 mM EDTA, 150 mM NaCl, and 0.5% sodium deoxycholate) as instructed (Beyotime, China). Supernatants of lysates were collected after centrifugation. A BCA protein detection kit (Beyotime, China) was used to determine the protein concentration. SDS/PAGE was used for the separation of the indicated amounts and then transferred on to PVDF membranes (Millipore, U.S.A.). Using nonfat milk, the membranes were blocked for 1 h. Next, primary antibodies were incubated overnight at 4°C. Then, the membranes were washed using TBST for 15 min and incubated with secondary antibodies (1:5000) at 37°C for 1 h. After washing with TBST for 15 min, the detection was performed with Fusion FX (Vilber, France) using an enhanced chemiluminescence kit (Millipore, U.S.A.). Specific bands were quantified using Fusion software. Each experiment was performed in triplicate. The following antibodies were used: CD133 (Proteintech, U.S.A.), CD44 (CST, U.S.A.), Lgr5 (Abcam, U.S.A.), EpCAM (Abcam, U.S.A.), ALDH1 (Abcam, U.S.A.), β-catenin (Proteintech, U.S.A.), CD166 (CST, U.S.A.), E-cadherin (CST, U.S.A.), N-cadherin (Epitomics, U.S.A.), vimentin (CST, U.S.A.), Snail (Abcam, U.S.A.), Twist (Abcam, U.S.A.), Slug (Abcam, U.S.A.), ZEB1 (Abcam, U.S.A.), fibronectin (Abcam, U.S.A.), and GAPDH (Goodhere, China).

### Transwell migration and invasion assay

For Transwell migration assays, positive and negative cells from the separation protocol were resuspended at a concentration of 1 × 10^4^ cells in 250 μl of DMEM-F12 and placed in the upper chamber with the noncoated membrane (Millipore, U.S.A.). Medium supplemented with 10% FBS was used as a chemoattractant in the lower chamber. After 24 h, the cells were fixed with 4% paraformaldehyde for 30 min and stained for 10 min in 0.5% Crystal Violet. Cells were counted under a light microscope at 100× magnification. The invasion assays were similar to the migration assays except using a top chamber with Matrigel-coated membrane (Millipore, U.S.A.).

### Assays for the adhesive capacity of cells to extracellular matrix proteins

To evaluate the adhesive capacity of different cell subsets to extracellular matrix (ECM) proteins, CD133^−^CD44^−^ and CD133^+^CD44^+^ cells were plated on to 60-mm dishes (5 × 10^5^ cells/dish) coated with type I collagen or fibronectin, respectively, and cultured for 12 h. Nonadherent and adherent cells were then collected and counted using a hemocytometer.

### Cell viability

5-fluorouracil (5-FU)- and oxaliplatin (OXLP)-induced toxicity was measured by functional impairment of the mitochondria using MTT methods (Sigma, U.S.A.). Approximately 5 × 10^4^ HT29-CD133^−^CD44^−^ and HT29-CD133^+^CD44^+^ cells per well were seeded in a 96-well plate and then cultured. Where indicated, cells were treated for 24 h with increasing concentrations of OXLP (up to 100 μM) (Sigma, U.S.A.) or with increasing concentrations of 5-FU (up to 500 μg/ml) (Sigma, U.S.A.). Ten microliters of 5 mg/ml MTT was directly added to the cells, followed by incubation for 4 h at 37°C. Then, the medium was centrifuged and removed, and 100 μl dimethyl sulfoxide (DMSO) was added to dissolve formazan crystals. A Synergy 2 Multi-Mode Microplate Reader was used to quantify the absorbance at 570 nm to determine the number of viable cells. Cell viability (%) was calculated as follows: (absorbance of test sample/absorbance of control) × 100%. All experiments were performed three times for each experiment.

### Assay for the measurement of intracellular reactive oxygen species

Intracellular reactive oxygen species (ROS) levels of HT29-CD133^+^CD44^+^ and HT29-CD133^−^CD44^−^ cells were measured. ROS were detected with a 2′,7′-dichlorofluorescein diacetate (DCFH-DA) probe (Beyotime, China) according to the manufacturer’s manuals. Briefly, cells were incubated with 10 μmol/l DCFH-DA at 37°C for 20 min. Then, cells were washed three times in PBS to remove the DCFH-DA that had not entered the cells, and then the cells were suspended in medium and immediately analyzed by flow cytometry.

### Sphere formation assay

Similar to previous studies [7], a sphere formation assay was performed. Isolated CD133^−^CD44^−^ and CD133^+^CD44^+^ cells (1 × 10^4^ cells/well) were plated on to six-well ultralow cluster plates (Corning, U.S.A.). Cells were cultured in serum-free DMEM-F12 medium (Gibco, U.S.A.) with 20 ng/ml EGF (Peprotech, U.S.A.), B27 supplement (1:50, Gibco, U.S.A.), and 20 ng/ml basic FGF (Peprotech, U.S.A.). The number of cell spheres was counted after 3 weeks of culture.

### Alkaline comet assay for assessing DNA damage

DNA damage was assessed by alkaline comet assay following: Singh et al.’s procedure with some modifications [20]. Briefly, single-cell suspensions of HT29-CD133^−^CD44^−^ and HT29-CD133^+^CD44^+^ cells with or without exposure to chemotherapy drugs were prepared. These cells were embedded in low-melting-point agarose and were lysed overnight at 4°C in lysis buffer (0.1 M EDTA, 1% Triton X-100, 0.01 M Tris base, 2.5 M NaCl, 5% DMSO, pH 10). Then, the unwinding step was performed in unwinding/electrophoresis buffer (0.3 M NaOH, 2 mM EDTA, pH 13) for 1 h at 4°C. Electrophoresis was conducted for 25 min at 4°C in unwinding/electrophoresis buffer at electric-field strengths of 306 mA and 0.6 V/cm. The slides were then neutralized with neutralizing buffer (0.4 Tris/HCl, pH 7.5), rinsed with distilled water, air-dried, stained with 20 µl Ethidium Bromide (2 µg/ml), and covered with standard cover slips. Sides were analyzed for comets with a Nikon Optiphot microscope attached to a Pulnix video camera and fluorescence image analysis system. DNA single-strand breaks were determined by the tail moment parameter.

### Microarray experiments and gene set enrichment analysis

Positive and negative cells were isolated from HCT116 and HT29 cell lines, and RNA was extracted for microarray experiments. HG-U133 Plus 2.0 microarrays were hybridized with 10 μg cRNA and processed per the manufacturer’s protocol (Affymetrix, U.S.A.). Three biological replicates from different cell samples were quantile normalized. Using the AFFY and GCRMA packages, hybridization signals were generated in BioConductor. Heatmaps and hierarchical clustering analysis were used to display the expression patterns of genes that are mechanistically involved in the Wnt/β-catenin signaling pathway, EMT process and CRC metastasis. Moreover, the expression of genes involved in ROS metabolism was investigated by gene set enrichment analysis (GSEA) as previously described [21,22]. In brief, using the Gene Ontology GO:TERMFINDER program, we initially generated a list of genes associated with ROS metabolism and regulation. Next, those genes with published evidence of involvement in ROS metabolism or regulation were retained. Finally, GSEA was applied to determine whether these ROS-related genes were randomly distributed or enriched at the top or bottom of the entire reference gene set.

### Statistical analyses

Data are expressed as the mean ± standard deviation. Statistical analyses were performed using unpaired two-tailed Student’s *t* test, paired *t* test, and one-way ANOVA with Newman–Keuls as post hoc test in GraphPad PRISM (San Diego, CA, U.S.A.). Differences with *P*<0.05 were considered statistically significant (*P<0.05; **P<0.01; ***P<0.001; ****P<0.0001), and n.s., no statistically significant difference (*P*≥0.05).

## Results

### Expression of CD133 and CD44 in colon cancer cell lines

**Figure 1:**
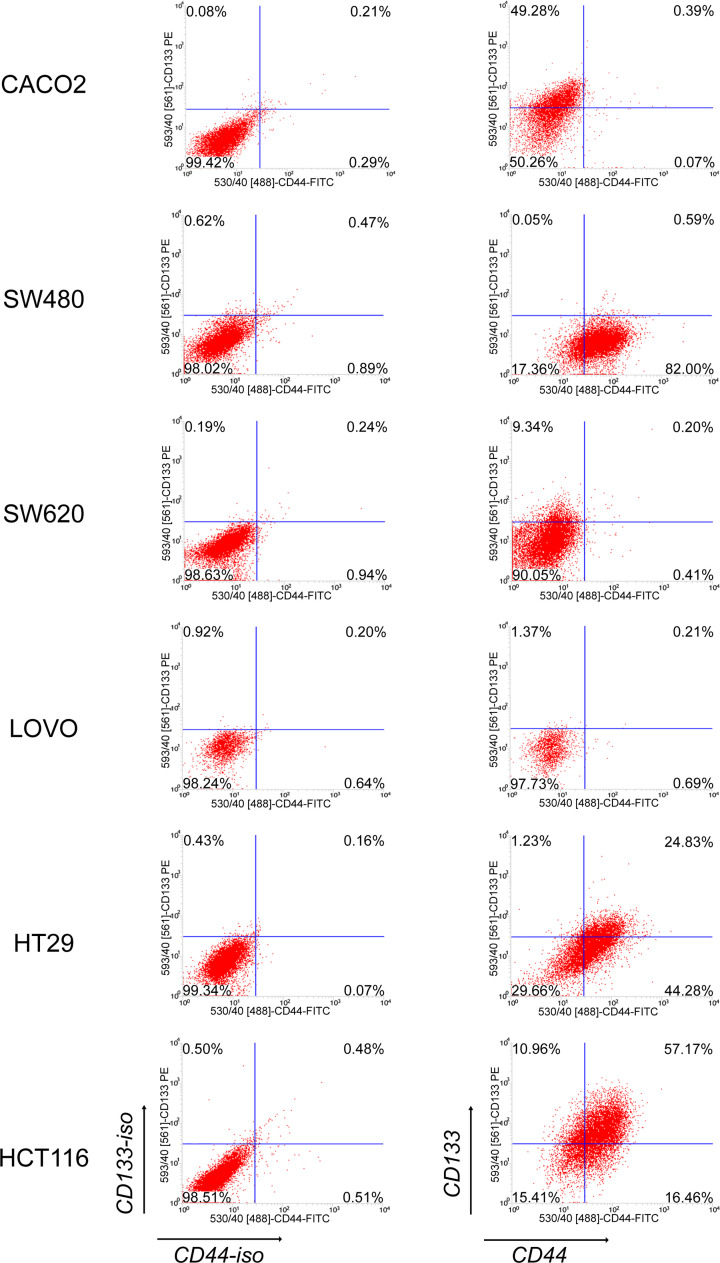
The expression of CD133 and CD44 in six human established colon cancer cell lines Cells were stained with FITC-labeled anti-CD44 and PE-labeled anti-CD133 antibodies and analyzed by flow cytometry. Isotype control was used to exclude nonspecial positive expression of CD133 and CD44. Representative dot plots are shown.

First, we aimed to identify putative LT-TIC populations, and a total of six well-characterized human colon cancer cell lines were used. LT-TICs were identified by using two surface markers, CD133 and CD44, which have been previously described as the most important markers for isolating colon CSCs [4,5,7,9]. CD133 and CD44 expression was detected by flow cytometry analysis, and the expression rate presented large differences in the six cell lines (Figure 1). CD133 was expressed by a majority of tumor cells in the HCT116 cell line (68%), whereas in the remaining cell lines, it was expressed either by a moderate proportion of tumor cells [as in the Caco2 (49%) and HT29 (26%) lines] or by a restricted cell subset [as in the Lovo (4%) and SW620 (9%) cell lines]. Finally, in SW480 cell lines, it was not expressed at all. CD44 was expressed by a majority of cells in the HT29 (69%), HCT116 (74%), and SW480 (82%) cell lines. In the Lovo cell line, however, CD44 expression was present only on a minor cell fraction (1.3%), and in the SW620 and Caco2 cell lines, it was completely negative. Notably, in most cell lines (SW480, Lovo, SW620, and Caco2), CD133 and CD44 were not coexpressed. However, in the HCT116 and HT29 cell lines, coexpression of CD133 and CD44 molecules was detected in a moderate cell proportion (Figure 1). Therefore, HT29 and HCT116 cell lines were used for further study.

### CD133^+^CD44^+^ cells functionally confirm the extensive tumorigenic potential of LT-TICs

**Figure 2:**
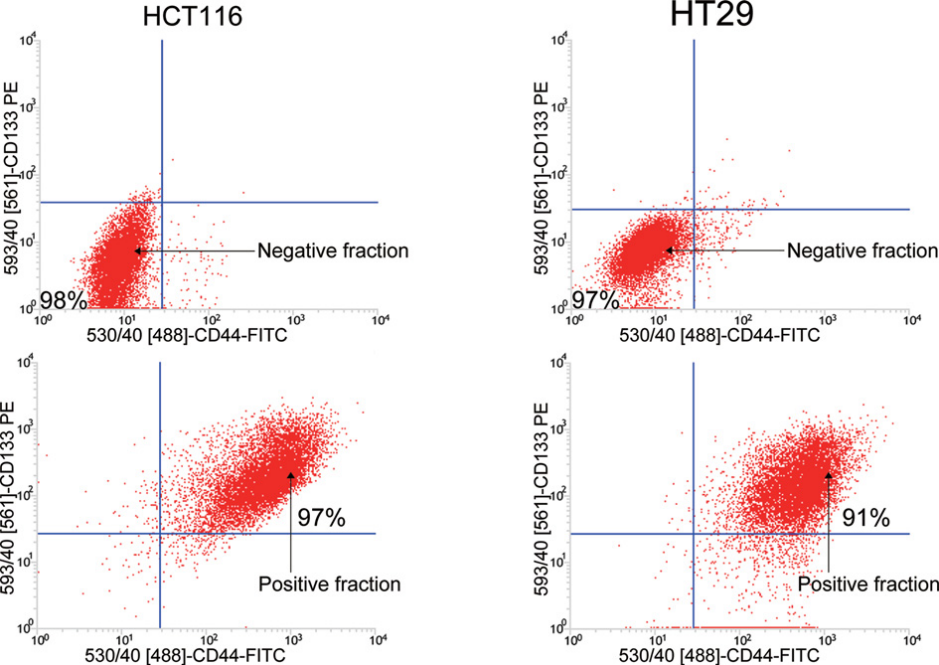
Isolation of CD133^+^CD44^+^ cells and CD133^−^CD44^−^ cells from HCT116 and HT29 cell lines CD133^+^CD44^+^ cells were isolated from HCT116 and HT29 cell lines by magnetic bead selection using the CD133 and CD44 antibodies labeled with MicroBeads. Samples were then stained with the PE-labeled anti-CD133 antibody and FITC-labeled anti-CD44 antibody, and analyzed by flow cytometry indicating excellent depletion for CD133^+^ and CD44^+^ cells in the negative fraction as well as high enrichment for CD133^+^CD44^+^ CSCs in the positive fraction. Representative dot plots are shown.

**Figure 3:**
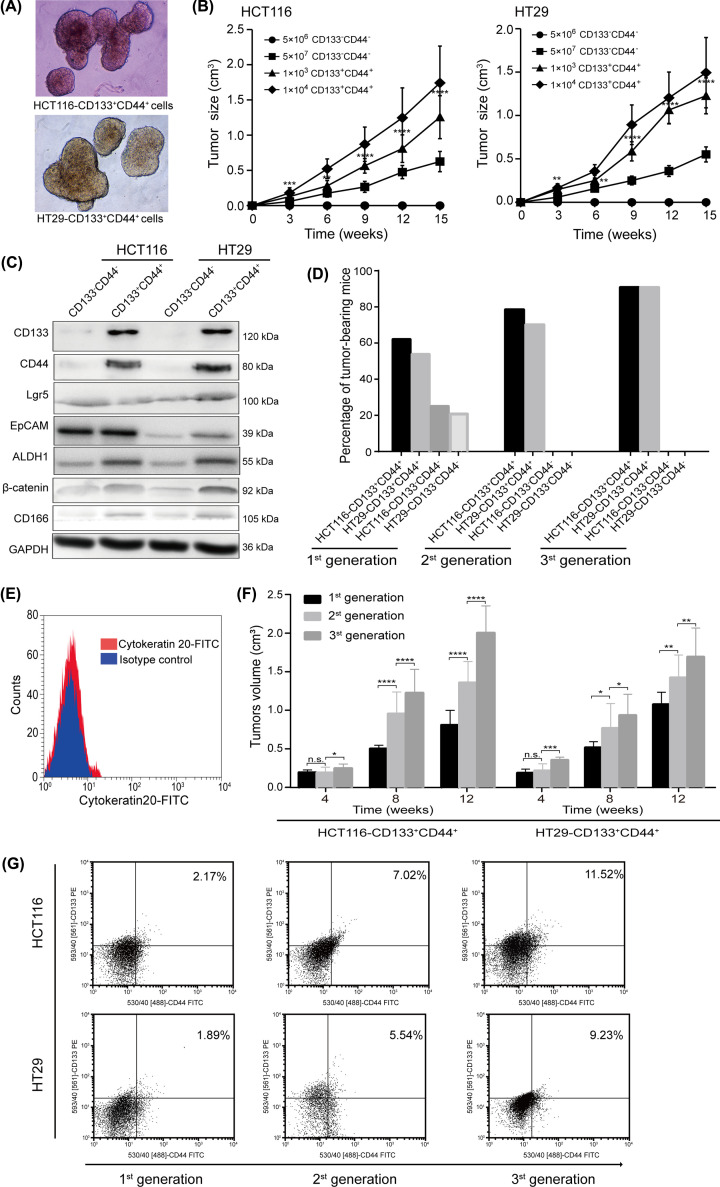
CD133^+^CD44^+^ CSCs functionally confirms the extensive tumorigenic potential of LT-TICs (**A**) Formation of spheres from CD133^+^CD44^+^ cells isolated from HCT116 and HT29 cell lines, respectively. (**B**) Tumorigenic potential of 1 × 10^3^ or 1 × 10^4^ CD133^+^CD44^+^ cells, and 5 × 10^6^ or 5 × 10^7^ CD133^−^CD44^−^ cells derived from HCT116 and HT29 cell lines, respectively. Xenografts were established from subcutaneous injection of different cell populations (24 injections per cell subsets). Data are presented as mean ± SD of tumor volume at different time points of different cell subsets. Statistical analysis: Student’s *t* test, **P<0.01, ***P<0.001, and ****P<0.0001, 1 × 10^3^ CD133^+^CD44^+^ cells vs. 5 × 10^7^ CD133^−^CD44^−^ cells. (**C**) Immunoblotting of colon cancer stem markers from lysates of CD133^+^CD44^+^ cells and CD133^−^CD44^−^ cells. CD133^+^CD44^+^ and CD133^−^CD44^−^ cells were isolated from HCT116 and HT29 cell lines, respectively. GAPDH is used as the loading controls. (**D**) After magnetic bead sorting of HCT116 and HT29 cells, 1 × 10^3^ CD133^+^CD44^+^ cells or 5 × 10^7^ CD133^−^CD44^−^ cells were subcutaneously implanted into 24 BALB/c nude mice in each group to induce the first-generation xenografts. Similarly, 1 × 10^3^ CD133^+^CD44^+^ cells or 5 × 10^7^ CD133^−^CD44^−^ cells from the first- and second-generation xenografts were again subcutaneously transplanted into secondary mice (*n*=24 each group) and three-generation mice (*n*=24 each group), respectively. The percentage of tumor-bearing mice in three-generation mice were calculated. (**E**) Flow cytometry analysis for cytokeratin 20 shows no expression in CD133^+^CD44^+^ cells dissociated from original xenografts. Experiments were performed in duplicates. (**F**) Tumor volumes of successive passages of xenografts at different time points. The first-generation xenografts were induced by subcutaneously injection of 1 × 10^3^ CD133^+^CD44^+^ cells isolated from HCT116 and HT29 cell lines, respectively. At the time of sacrifice of the first-generation recipients, tumors were dissociated and equivalent number of CD133^+^CD44^+^ cells implanted into secondary recipients followed by tertiary recipients. Data represent the mean ± SD of tumor volume. The number of tumors formed by each cell subsets are shown in (F). Statistical analysis: one-way ANOVA, Newman–Keuls as post hoc test, n.s., *P*≥0.05, *P<0.05, **P<0.01, ***P<0.001, and ****P<0.0001. (**G**) Flow cytometry analysis for the percentage of CD133^+^CD44^+^ cells in the first-, second-, third-generation isolated xenografts.

LT-TICs are characterized by their extensive self-renewal capacity, which initiated tumor formation [8]. Thus, we investigated the tumorigenic capacity of CD133^+^CD44^+^ cells isolated from HCT116 and HT29 cells. Specifically, CD133^−^CD44^−^ cells and CD133^+^CD44^+^ cells were isolated by magnetic bead sorting based on CD133- and CD44-labeled microbeads, which resulted in considerable positive enrichment of CD133^+^CD44^+^ cells (purity > 90%) and highly effective negative selection (purity > 97%) of CD133^−^CD44^−^ cells (Figure 2), as detected by flow cytometry analysis with staining of CD133 and CD44. Self-renewal capacity is widely evaluated by sphere formation assays [4,23,24]. We first examined the ability of CD133^+^CD44^+^ cells to form spheres *in vitro*. Within 3 weeks of serum-free culture, CD133^+^CD44^+^ cells from HT29 and HCT116 cell lines formed very large and integrated spheres (Figure 3A), whereas CD133^−^CD44^−^ cells almost completely died in such culture conditions (data not shown). Then, the *in vivo* tumorigenic potential of CD133^+^CD44^+^ cells was also assessed using a mouse subcutaneous xenograft model. As many as 5 × 10^6^ HCT116- or HT29-CD133^−^CD44^−^ colon cancer cells did not induce tumor formation, but as few as 1 × 10^3^ CD133^+^CD44^+^ cells generated visible tumors after 4 weeks (Figure 3B). These data suggest that the subset of cells capable of initiating colon tumors is highly enriched in CD133^+^CD44^+^ cells. Interestingly, we also found that tumors began to grow when up to 5 × 10^7^ HCT116- or HT29-CD133^−^CD44^−^ cells were subcutaneously injected into nude mice (Figure 3B). Notably, despite the large number of tumor cells in the negative cell population, the tumor formation rate and efficiency of 1 × 10^3^ purified CD133^+^CD44^+^ cells were higher than those of 5 × 10^7^ CD133^−^CD44^−^ cells (Figure 3B,D). To further understand the cause of the low tumorigenesis ability of such a large number of cells from the negative population, the protein expression levels of putative CSC markers, including CD133 [4,5,7], CD44 [9,19], CD166 [9,25], Lgr5 [26], EpCAM [27], ALDH1 [28] and β-catenin [29], in CD133^+^CD44^+^ cells and CD133^−^CD44^−^ cells were detected using Western blotting. We found that although CD133 and CD44 were not expressed in the CD133^−^CD44^−^ cell population, some other stem cell markers were expressed in CD133^−^CD44^−^ cells (Figure 3C); thus, the low tumorigenic potential of the very large negative cell population may be due to the incomplete clearance of the other CSC markers in the negative cell subset. Indeed, due to the lack of unique colon CSC surface markers, it seems impossible to isolate cancer-specific CSCs, even though more markers are used in stem cell sorting [30]. However, the results indicating that most of the putative CSC markers were significantly overexpressed in CD133^+^CD44^+^ cells compared with CD133^−^CD44^−^ cells (Figure 3C) further emphasized the powerful tumorigenic capacity of CD133^+^CD44^+^ cells.

**Figure 4:**
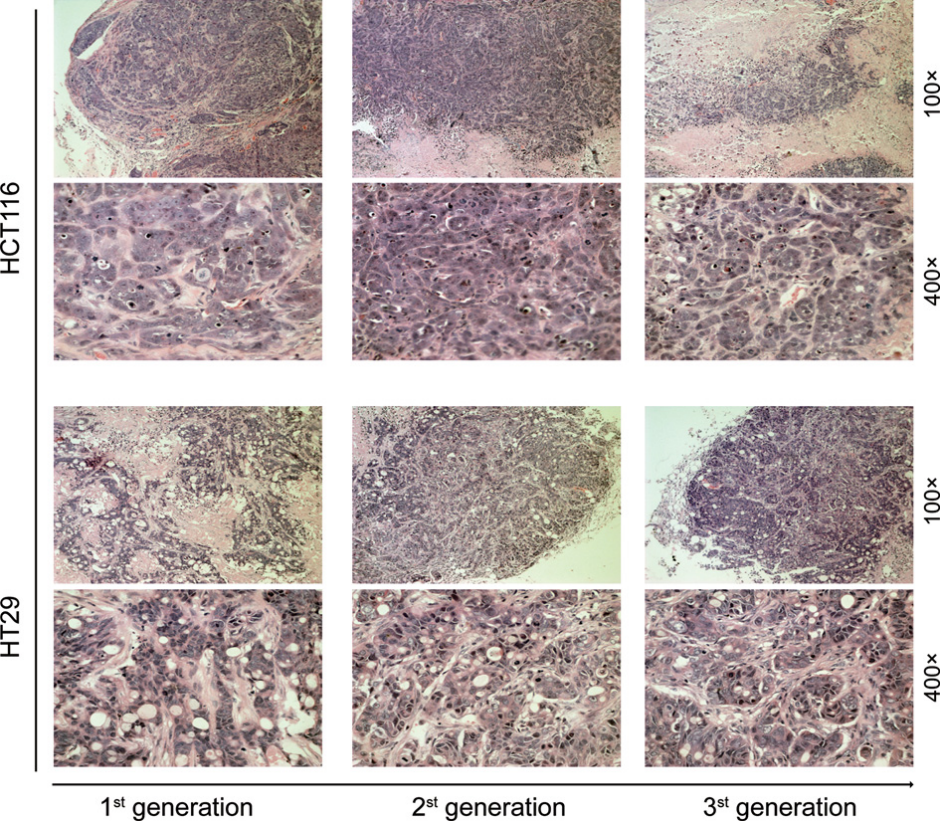
H&E staining for three generation tumor xenografts H&E staining of xenograft passages shows the three generation tumor xenografts derived from HCT116 and HT29-CD133^+^CD44^+^ cells presented the same histopathology features, respectively.

To investigate whether CD133^+^CD44^+^ cells have long-term tumorigenic potential, a critical functional feature of LT-TICs [8], we evaluated the capacity of these cells to induce tumors after serial transplantation. Tumor xenografts derived by the injection of purified HCT116- and HT29-CD133^+^CD44^+^ cells were digested to isolate CD133^−^CD44^−^ and CD133^+^CD44^+^ cells, and 1 × 10^3^ CD133^+^CD44^+^ cells or 5 × 10^7^ CD133^−^CD44^−^ cells from the first-generation xenografts were subcutaneously transplanted into secondary mice. Freshly dissociated CD133^+^CD44^+^ cells from original xenografts did not express cytokeratin 20 (CK20) (Figure 3E), an intermediate filament protein that was mainly restricted to intestinal epithelial cell differentiation [31]. Moreover, although 5 × 10^7^ HCT116- and HT29-CD133^−^CD44^−^ cells displayed limited capacity to form first-generation xenografts (Figure 3B,D), the equivalent cell number of CD133^−^CD44^−^ cells isolated from first-generation xenografts entirely failed to induce tumor formation in secondary recipients (Figure 3D). In contrast, 1 × 10^3^ CD133^+^CD44^+^ cells from the first-generation xenografts maintained their tumorigenic potential and were able to transfer the tumor into secondary mice (Figure 3D), confirming the data obtained with CD133^+^CD44^+^ cells directly isolated from cell lines. In addition, CD133^+^CD44^+^ cells obtained from similar CD133^+^CD44^+^-derived secondary xenografts were subsequently transplanted into third-generation mice. In the production of *in vivo* passages, we found that CD133^+^CD44^+^ cells did not lose their tumorigenic potential but instead had enhanced aggressiveness, as indicated by faster tumor growth and an increasing number of CD133^+^CD44^+^ cells in newly generated tumors (Figure 3F,G). Furthermore, the third-generation tumor xenografts derived from HCT116 or HT29-CD133^+^CD44^+^ cells presented the same histopathological features (Figure 4). Overall, CD133^+^CD44^+^ cells are able to generate serial xenografts showing virtually unlimited growth potential, which functionally confirms the extensive tumorigenic potential of LT-TICs.

### CD133^+^CD44^+^ cells also functionally reproduce the LT-TICs that drive TM formation

**Figure 5:**
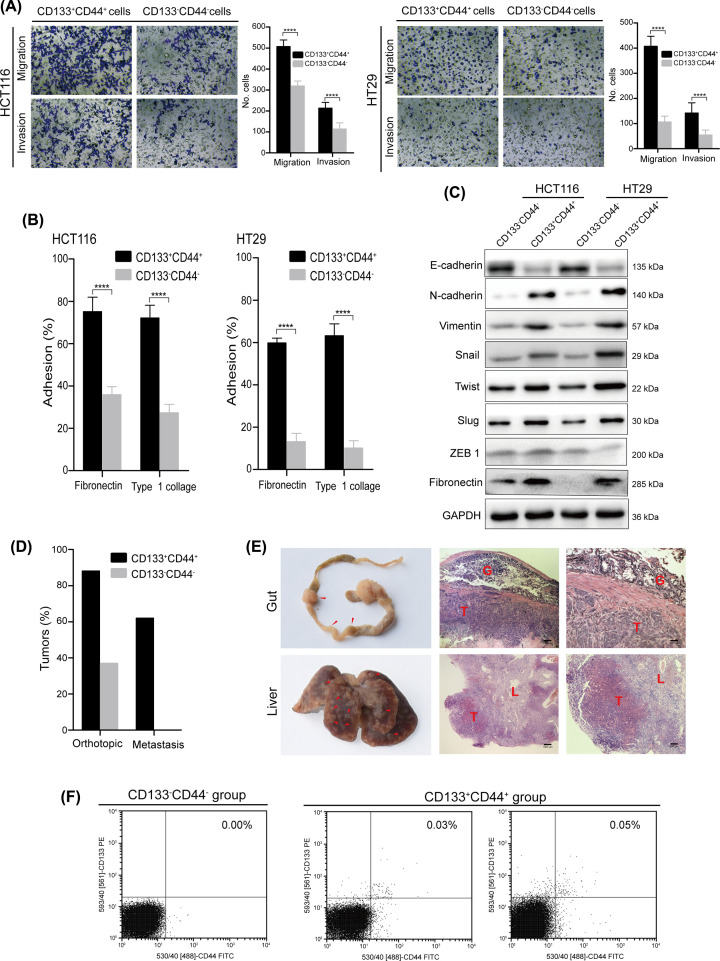
CD133^+^CD44^+^ CSCs also functionally reproduces the LT-TICs that drives TM formation (**A**) Invasive and migratory capacities of CD133^+^CD44^+^ and CD133^−^CD44^−^ cells isolated from HCT116 and HT29 cell lines, respectively. Bars represent the mean ± SD of invaded/migrated cells of three independent experiments in duplicate. Statistical analysis: Student’s *t* test, ****P<0.0001. (**B**) Adhesive capacity of CD133^+^CD44^+^ cells and CD133^−^CD44^−^ cells to the fibronectin or type 1 collagen, respectively. Percent adhesion is calculated as the number of adhesive cells/adhesive cells+nonadhesive cells. Data are expressed as the percent of adhesive cells determined over three fields per assay and expressed as an average of triplicate determinations. Statistical analysis: Student’s *t* test, ****P<0.0001. (**C**) Immunoblotting of EMT proteins from lysates of CD133^+^CD44^+^ cells and CD133^−^CD44^−^ cells isolated from HCT116 and HT29 cell lines, respectively. GAPDH is used as the loading controls. (**D**) Percentage of colon tumor and liver metastasis growth after 14–18 weeks in mice (*n*=16 per group) orthotopically injected with 1 × 10^4^ CD133^+^CD44^+^ cells and 5 × 10^7^ CD133^−^CD44^−^ cells. (**E**) Macroscopic analysis (left) and H&E staining (central) with corresponding magnifications (right) of tumors grown in the gut and liver after orthotopic injection of 1 × 10^4^ CD133^+^CD44^+^ cells. Gut (G), liver (L), and tumors (T) are indicated. Red arrowheads emphasize tumor multifocal dissemination along the gut and metastatic foci of liver. (**F**) Portal vein blood was used to investigate whether the circulation blood contain circulating migrating CSCs characterized by double staining for CD133 and CD44. CD133^+^CD44^+^ group and CD133^−^CD44^−^ group indicate those mice which were orthotopically injected with 1 × 10^4^ CD133^+^CD44^+^ cells and 5 × 10^7^ CD133^−^CD44^−^ cells, respectively. Flow cytometry shows a rare circulating CD133^+^CD44^+^ CSCs in the portal vein blood from mice at week 10 after orthotopic implantation of CD133^+^CD44^+^ cells isolated from HCT116 and HT29 cell lines, respectively.

In addition to their extensive tumorigenic potential, another critical function of LT-TICs in colon cancer is to drive TM [8]. Thus, based on the functional role of CD133^+^ and CD44^+^ cells in colon cancer (see introduction), we hypothesized that, similar to the functional characteristics of LT-TICs, CD133^+^CD44^+^ cells also possessed the capacity to drive TM. To test this hypothesis, we first aimed to investigate the migration and invasion ability of CD133^+^CD44^+^ cells. Our Transwell results revealed that CD133^+^CD44^+^ cells had much higher invasion and migratory capacities than CD133^−^CD44^−^ cells (Figure 5A). Furthermore, as tumor cell adhesion to the ECM is essential for cancer invasiveness [32], we tested the invasiveness of CD133^+^CD44^+^ cells in binding to the ECM. Indeed, the ability of CD133^+^CD44^+^ cells to adhere to both type 1 collagen and fibronectin was higher than that of CD133^−^CD44^−^ cells (Figure 5B). The invasion capacity of tumor cells is often related to the EMT of cells, which involves the loss of cell–cell interactions together with the acquisition of migratory properties [33]. We therefore tested the expression of EMT regulatory proteins in CD133^+^CD44^+^ versus CD133^−^CD44^−^ cells. Our results showed that the expression of the epithelial marker E-cadherin was down-regulated in CD133^+^CD44^+^ cells compared with CD133^−^CD44^−^ cells (Figure 5C). Moreover, concomitant up-regulation of the mesenchymal markers N-cadherin and vimentin, other EMT markers, such as snail, twist, slug, ZEB1, and fibronectin, was also up-regulated in CD133^+^CD44^+^ cells (Figure 5C). These *in vitro* findings indicate that EMT-like attributes contribute to the migratory capacity and the invasive phenotype of CD133^+^CD44^+^ cells.

**Figure 6:**
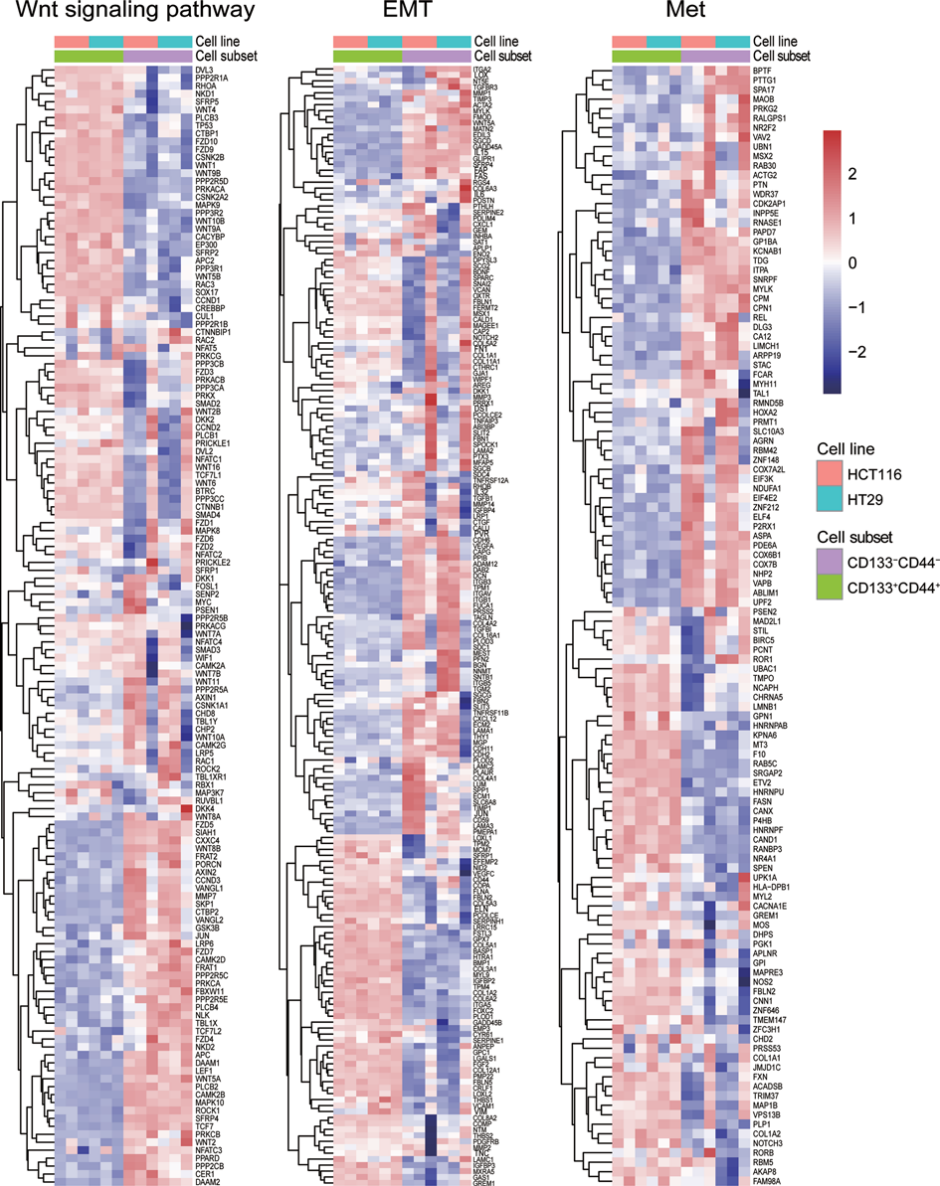
Microarray analysis of up-regulated and down-regulated Wnt-, EMT-, and TM (Met)-related genes in CD133^+^CD44^+^ and CD133^−^CD44^−^ cells

Based on this *in vitro* evidence, in a further step, we aimed to test the *in vivo* metastatic capacity of CD133^+^CD44^+^ cells. For this purpose, 5 × 10^7^ CD133^−^CD44^−^ cells and 1 × 10^4^ CD133^+^CD44^+^ cells were injected into the mouse cecal wall. Orthotopic implantation of 5 × 10^7^ CD133^−^CD44^−^ cells generated small tumors compared with CD133^+^CD44^+^ cells, and these tumors were confined to the cecum (Figure 5D). However, 1 × 10^4^ CD133^+^CD44^+^ cells not only initiated tumor growth in the colorectum but also robustly generated metastatic lesions in the gut and liver after 14–18 weeks (Figure 5D,E). These data suggest that only 1 × 10^4^ CD133^+^CD44^+^ cells can form both orthotopic tumors and metastases, whereas a large number of CD133^−^CD44^−^ cells grew locally as small tumors without forming distant lesions. The process of distant metastasis involves circulating CSCs [34]. Thus, we detected circulating CD133^+^CD44^+^ cells in the portal vein of mice at week 10 after cecal wall injection. Flow cytometric analysis identified a reproducible population of circulating CD133^+^CD44^+^ CSCs only in the CD133^+^CD44^+^ group (Figure 5F), demonstrating the invasion of CD133^+^CD44^+^ cells into the circulation; no circulating CD133^+^CD44^+^ cells were detected in mice orthotopically implanted with CD133^−^CD44^−^ cells (Figure 5F). To analyze the subpopulation of CD133^+^CD44^+^ cells with metastatic capacity in more detail, microarray analysis of the CD133^+^CD44^+^ and CD133^−^CD44^−^ tumor cells was performed; the cells were clustered into two separate subgroups, and the results showed that the expression of Wnt-, EMT-, and TM-related genes was significantly changed in the CD133^+^CD44^+^ subset (Figure 6). Taken together, these findings suggest that CD133^+^CD44^+^ cells are able to drive TM, in line with LT-TICs at the functional level. In summary, similar to the methods for isolating and identifying CSCs and on the basis of the two critical functions of LT-TICs, which extensively self-renew and readily metastasize, here, we provide multiple lines of evidence that CD133^+^CD44^+^ cells not only functionally confirm the extensive tumorigenic potential of LT-TICs but also functionally reproduce the LT-TICs that drive TM. These data indicate that CD133^+^CD44^+^ cells are enriched in LT-TICs and can act as a typical representation of LT-TICs in colon cancer.

### Resistance of CD133^+^CD44^+^ cells to chemotherapy

**Figure 7:**
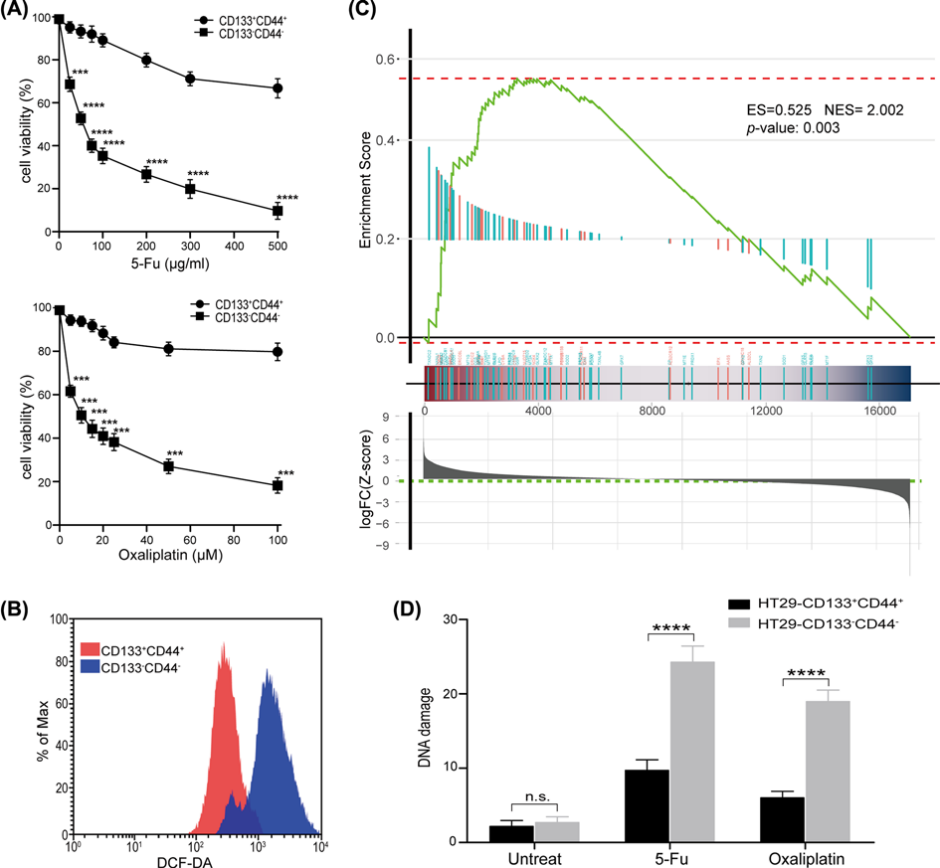
Resistance of LT-TICs (CD133^+^CD44^+^ CSCs) to chemotherapy (**A**) Effect of 24-h exposure of CD133^+^CD44^+^ and CD133^−^CD44^−^ cells isolated from HT29 cells to different concentrations of 5-FU or OXLP. Data are presented as mean ± SD of three independent experiments with triplicates for each dose. Statistical analysis: Student’s *t* test, ***P<0.001, and ****P<0.0001 vs. CD133^+^CD44^+^ cells. (**B**) CD133^+^CD44^+^ and CD133^−^CD44^−^ cells were isolated from HT29 cells and intracellular ROS concentrations were measured by flow cytometry with DCFH-DA staining. (**C**) Expression of ROS genes in colon cancer subpopulations. GSEA was used to analyze the enrichment of a curated list of genes involved in ROS metabolism using microarray gene expression data from sorted CD133^+^CD44^+^ and CD133^−^CD44^−^ cells. The GSEA enrichment plot is shown. *P=*0.003. (**D**) CD133^+^CD44^+^ and CD133^−^CD44^−^ cells were isolated from HT29 cells and exposed with 500 μg/ml 5-FU or 100 μM OXLP. DNA damage was measured before drug exposure and 24 h later after drug exposure using the alkaline comet assay. Data are presented as mean of median tail moments ± SD of three independent experiments in triplicates. Statistical analysis: Student’s *t* test, n.s., *P*≥0.05, and ****P<0.0001.

Although we have identified that LT-TICs possess self-renewal and metastasis-driving potential, their chemoresistance is unknown. Thus, we analyzed the cell viability of CD133^+^CD44^+^ cells and CD133^−^CD44^−^ cells dissociated from HT29 cell lines following exposure to 5-FU or OXLP at clinically relevant doses. Our data demonstrated that CD133^−^CD44^−^ cells presented high *in vitro* sensitivity to both drugs tested in a dose-dependent fashion (Figure 7A). In contrast, CD133^+^CD44^+^ cells were largely resistant to chemotherapeutic drug-induced apoptosis even at the highest dose concentrations used (Figure 7A). These data indicate that chemotherapeutic treatments fail to eliminate LT-TICs, which expands the functional definition of LT-TICs.

Recent studies have demonstrated that compared with non-CSCs, CSCs in some tumors contain significantly lower concentrations of ROS, which are essential for the resistance of cells to radiation and chemotherapy [35,36]. Thus, we hypothesized that LT-TICs in colon cancer also contain low ROS levels. To test this hypothesis, intracellular ROS levels in CD133^−^CD44^−^ cells and CD133^+^CD44^+^ cells were measured by flow cytometry with staining of DCFH-DA. Our results revealed that the CD133^+^CD44^+^ cells displayed lower ROS levels than CD133^−^CD44^−^ cells (Figure 7B). Moreover, to evaluate ROS biology in LT-TICs, GSEA was performed. The results revealed that ROS metabolism genes were highly overrepresented in the CD133^+^CD44^+^ cells (Figure 7C), and the ROS genes identified as the core enriched genes by GSEA included a number of important antioxidant genes (Table 1), which partly explained the lower ROS levels in LT-TICs. Since cell death after exposure to cytotoxic chemotherapeutics is partially mediated by DNA damage [37] and given our observations of decreased levels of ROS and enhanced ROS defense gene expression in LT-TICs, we were therefore interested in testing whether CD133^+^CD44^+^ cells develop less DNA damage after exposure to OXLP or 5-FU than CD133^−^CD44^−^ cells. The alkaline comet assay showed that untreated cells did not display significantly different levels of DNA damage, while fewer DNA-strand breaks were observed in the CD133^+^CD44^+^ cells after exposure to OXLP or 5-FU (Figure 7D). Thus, consistent with the lower levels of ROS and the enhanced expression of antioxidant genes involved in ROS defense in CD133^+^CD44^+^ cells, these positive cells developed fewer DNA-strand breaks than CD133^−^CD44^−^ cells after exposure to chemotherapeutics.

**Table 1:** GSEA leading edge analysis identified the indicated genes as being the core enriched genes which included a number of important antioxidant genes

Symbol	Gene ID	Gene description	Probe_id
CCS	9973	copper chaperone for superoxide	203522 at
GLRX2	51022	glutaredoxin 2	219933 at
GLRX3	10539	glutaredoxin 3	207506 at
GPX1	2876	glutathione peroxidase 1	200736 s at
GSR	2936	glutathione reductase	205770 at
LPO	4025	lactoperoxidase	210682 at
MPO	4353	myeloperoxidase	203948 s at
MSRA	4482	methionine sulfoxide reductase A	219281 at
MT1G	4495	metallothionein 1G	204745 × at
MT1H	4496	metallothionein 1H	206461 × at
MT1M	4499	metallothionein 1M	217546 at
MT2A	4502	metallothionein 2A	212185 × at
NXNL1	115861	nucleoredoxin-like 1	1553755 at
PRDX2	7001	peroxiredoxin 2	201006 at
PRDX4	10549	peroxiredoxin 4	201923 at
PRDX6	9588	peroxiredoxin 6	200844 s at
SOD3	6649	superoxide dismutase 3, extracellular	205236 × at
TXNDC11	51061	thioredoxin domain containing 11	223325 at
TXNDC17	84817	thioredoxin domain containing 17	224511 s at
TXNDC2	84203	thioredoxin domain containing 2	1552657 a at
TXNDC8	255220	thioredoxin domain containing 8	1564386 at
TXNDC9	10190	thioredoxin domain containing 9	1554047 at
TXNL1	9352	thioredoxin-like 1	201588 at
TXNL4A	10907	thioredoxin-like 4A	1555461 at
TXNRD1	7296	thioredoxin reductase 1	1561080 at
TXNRD2	10587	thioredoxin reductase 2	210803 at

## Discussion

All differentiated colon epithelial cells are produced by pluripotent stem cells, which are located in the basis of the colon epithelium and have unlimited proliferative potential [38,39]. Owing to the short lifespan of terminally differentiated cells, the stem cell system has to generate millions of differentiated colonocytes in the lifetime of each stem cell [40]. Thus, to quickly adapt to changes in cell needs in a controlled manner, distinct stem cell and progenitor cell types are hierarchically organized [26,41]. In each colon crypt, there are only four to six long-lived stem cells with an infinite capacity to proliferate, and they produce transient amplifying progenitor cells to move their progeny up the crypt walls [42–44]. In addition, these progenitor cells give rise to hundreds of differentiated cells shedding from the top of villi every 4–5 days [45].

Human tumors are maintained by CSCs formed from normal stem/progenitor cells with the characteristics of malignancy [10,11]. In cancers caused by genetic or inherited mutations, molecular damage most likely occurs in long-lived specialized tissue stem cells with unlimited self-renewing potential [46–48]. There is growing evidence that stem cell-like properties can be tested in distinct cells of colonic epithelial malignancies [4,5,9,25,49]. In immunodeficient mice, a small number of TICs proved to be the only ones capable of forming tumors [4,5,9]. It has been estimated that the frequency of TIC reactions presents wide differences in different cancers and cancer entities [50–52]. Compared with highly malignant melanoma, colon cancer TICs have proven to be rare and associated with a distinct phenotype [4,5,50]. Accumulating evidence indicates that TICs have the same key biological characteristics as normal stem cells [9,50]. They are thought to be the source of tumorigenic and nontumorigenic cell regeneration, but molecular evidence of their self-renewal ability is still lacking. Recently, Dieter et al. found unexpected cellular heterogeneity in the TIC compartment of colon cancer using a molecular tracking strategy [8]. They suggested that LT-TICs are responsible for the tumorigenesis and metastasis of colon cancer and are a reliable target for cancer radical treatment [8]. However, there is no specific way to isolate and identify LT-TICs. In the present study, based on the methods for isolating stem cells, we isolated and identified a subset of CD133^+^CD44^+^ cells that may be enriched in LT-TICs of colon cancer for the first time.

Human cancers have a large amount of heterogeneity in terms of protein and gene expression, tumor growth, metastatic properties, and chemoresistance [53–55]. Currently, on the basis of putative cell surface markers for embryonic, normal and malignant stem cells, TICs can be effectively identified and isolated from a variety of tumors and established cell lines. Then, these isolated cell subsets were confirmed by the characteristics of TICs for tumor initiation and phenotypic plasticity [56–58]. In our study, a total of six colon cancer cell lines were used. Coexpression of CD133 and CD44 was detected in a majority of cells in the HCT116 and HT29 cell lines, which is in line with previous evidence that colon cancer cell lines contain the TIC cell-like subset [59]. Growing evidence has demonstrated that LT-TICs present extensive tumorigenic potential and drive TM [8]. Thus, to effectively isolate LT-TICs, the candidate cell surface marker has to confirm these two important phenotypes of LT-TICs. Previous studies have widely confirmed that CD133 has a powerful ability to initiate colon cancer growth but lacks the capacity for TM [12–14]. In contrast, CD44 is thought to be extensively involved in the process of colon cancer metastasis, but it seems to not present long-term tumorigenic potential [18,19]. Thus, we speculate that CD133 and CD44 may be ideal markers for isolating LT-TICs. In the present study, our data showed that CD133^+^CD44^+^ cells resident in the colon tumor mass are able to generate serial xenografts showing virtually unlimited growth potential, which is in line with the critical functional feature that LT-TICs maintain tumor formation in serial xenotransplants. Moreover, we also provide several lines of evidence to confirm that CD133^+^CD44^+^ cells are responsible for the metastasis of colon cancer, another critical functional characteristic of LT-TICs. Mechanistically, the enhanced capacity of CD133^+^CD44^+^ cells to drive metastasis involves up-regulated expression of Wnt-, EMT-, and metastasis-related genes in these cells. These findings suggest that the CD133^+^CD44^+^ cell subset is a typical representation of LT-TICs in colon cancer. More importantly, we further explored the functional definition of LT-TICs as significant chemoresistance was also observed in CD133^+^CD44^+^ cells. In addition, our results indicate that the root cause of the chemoresistance of colon cancer may be the higher levels of antioxidant genes involved in antioxidant defense systems and the lower levels of ROS in CD133^+^CD44^+^ cells.

In summary, due to the lack of unique or special CSC markers, the colon CSC model has been widely questioned, and controversy about the well-demonstrated colon CSC markers, including CD133 and CD44, has also been widely discussed. Indeed, in the present study, although multiple lines of evidence demonstrated that CD133^+^CD44^+^ cells are enriched in LT-TIC populations, whether these cell populations are real colon CSCs needs to be more accurately defined in further studies. However, the present study provides a basis for future studies on colon CSCs or LT-TICs. Furthermore, a potential treatment strategy targeted at regulating LT-TICs may have important clinical implications for eradicating colon cancer.

## Abbreviations

5-FU: 5-fluorouracil
ALDH1: aldehyde dehydrogenase, cytosolic 1-like
CSC: cancer stem cell
DCFH-DA: 2′,7′-dichlorofluorescein diacetate
DMSO: dimethyl sulfoxide
ECM: extracellular matrix
EMT: epithelial–mesenchymal transition
FBS: fetal bovine serum
FGF: fibroblast growth factor
FITC: fluorescein isothiocyanate
GAPDH: glyceraldehyde-3-phosphate dehydrogenase
GSEA: Gene Set Enrichment Analysis
H&E: hematoxylin-eosin staining
LT-TIC: long-term tumor-initiating cell
MACS: magnetic activated cell sorting
OXLP: oxaliplatin
PE: phycoerythrin
ROS: reactive oxygen species
TIC: tumor-initiating cell
TM: tumor metastasis
ZEB1: zinc finger E-box binding homeobox 1
